# Condition Monitoring of Railway Bridges Using Vehicle Pitch to Detect Scour

**DOI:** 10.3390/s24051684

**Published:** 2024-03-05

**Authors:** Claire McGeown, David Hester, Eugene J. OBrien, Chul-Woo Kim, Paul Fitzgerald, Vikram Pakrashi

**Affiliations:** 1 School of Civil Engineering, University College Dublin, D04 V1W8 Dublin, Ireland; eugene.obrien@ucd.ie (E.J.O.); paul.fitzgerald.3@ucdconnect.ie (P.F.); 2 School of Natural and Built Environment, Queen’s University Belfast, Belfast BT9 5AJ, UK; d.hester@qub.ac.uk; 3 Department of Civil and Earth Resources Engineering, Kyoto University, Kyoto 615 8540, Japan; kim.chulwoo.5u@kyoto-u.ac.jp; 4 UCD Centre for Mechanics, Dynamical Systems and Risk Laboratory, School of Mechanical and Materials Engineering, University College Dublin, D04 V1W8 Dublin, Ireland; vikram.pakrashi@ucd.ie; 5 SFI Centre for Energy, Climate and Marine Research and Innovation (MaREI), University College Dublin, D04 V1W8 Dublin, Ireland

**Keywords:** drive-by, indirect, bridge, Structural Health Monitoring, rotation, pitch, scour, erosion

## Abstract

This study proposes the new condition monitoring concept of using features in the measured rotation, or ‘pitch’ signal, of a crossing vehicle as an indicator of the presence of foundation scour in a bridge. The concept is explored through two-dimensional vehicle–bridge interaction modelling, with a reduction in stiffness under a pier used to represent the effects of scour. A train consisting of three 10-degree-of-freedom carriages cross the model on a profiled train track, each train varying slightly in terms of mass and velocity. An analysis of the pitch of the train carriages can clearly identify when scour is present. The concept is further tested in a scaled laboratory experiment consisting of a tractor–trailer crossing a four-span simply supported bridge on piers. The foundation support is represented by four springs under each pier, which can be replaced with springs of a reduced stiffness to mimic the effect of scour. The laboratory model also consistently shows a divergence in vehicle pitch between healthy and scoured bridge states.

## 1. Introduction

Bridges are a critical component of the infrastructure that supports national and local economies, aiding the transportation of people and goods and connecting communities. Scour is defined by Hamill [1] as the removal of soil from around bridge foundations by the action of flowing water and is the leading cause of bridge failure in the UK and the US [2,3]. It is anticipated that bridges over waterways will face an increased risk of scour erosion in the future due to the increased frequency of extreme weather as a result of climate change [4,5].

In most developed countries, bridge condition is predominately assessed by visual inspections [6], and for bridge piers situated in deep waterways, this is carried out by qualified sub aqua divers. The visibility of the foundations is often restricted because of murky water, and scour is hard to detect, even in shallow waterways, as often the scour holes infill with sediment, which has weaker mechanical properties than the original riverbed. For example, the sudden collapse of Malahide Bridge, Dublin, Ireland, in 2009 occurred three days after an inspection did not identify scour which was present at the time [7]. Scour can lead to the rapid compromise of foundation stiffness and the catastrophic collapse of the bridge, and in extreme circumstances, loss of life [8,9]. As a result, bridges identified as a scour risk are required to undergo much more frequent visual inspections. In Cumbria, UK, after intensive rainfall in 2015, many bridges were classified as having a high scour risk and requiring emergency inspections. Underwater inspections were delayed for many bridges due to health and safety concerns associated with working during a flood event and also by the limited number of qualified divers. Full inspections for over 180 bridges were delayed until the autumn of the next year [5,10]. For these reasons, amongst others, there is considerable research interest in the development of remote electronic sensing systems.

There are many fixed instrumentation devices which aim to detect scour. These are normally installed at a high-risk scour location in the vicinity of a bridge. Float-out devices are buried or driven into the riverbed. A response in a data logger installed on the bridge or nearby is triggered when the riverbed is eroded to a point which causes the device to float out and change position. These devices need to be reburied once they have been triggered and as such do not offer continuous monitoring [11,12]. The principle of a magnetic sliding collar device is that, as scour develops, the magnetic collar will slide down the rods which have been driven into the riverbed, allowing a reading to be taken of the collar’s new position [13]. Smart rocks are magnets encased in concrete and embedded upstream of the bridge which roll into scour holes when they have been eroded from the riverbed, with the relocation detected from a sensor mounted nearby [14,15,16]. Driven rod systems are embedded into the riverbed and the length of the exposed rod is back-calculated from the change in natural frequency of the rod using fibre-optic Gragg grating sensors, triggering an alert if scour has occurred. Other driven rod systems detect the increase in strain activity when scour exposes the embedded sensors to flowing water [17,18,19]. Dielectric probes can be inserted into the riverbed in a scour risk zone, measuring the electromagnetic properties of the soil. These can detect the changing bed level of a river as well as the difference between in situ and redeposited material in a scour hole [20,21]. Time-Domain Reflectometry systems also monitor the dielectric permittivity of the riverbed but in relation to a fixed probe [22,23]. A limitation of these fixed instrumentation devices (including, but not limited to, those described above) is that installation is required on the riverbed, and that they are only capable of detecting scour in the vicinity of the device.

A number of systems have been developed that use electromagnetic waves, sound waves, and satellite radar to monitor the progression of scour. Some of these devices are completely mobile and deployed on lorry-mounted cranes or unmanned boats. Known issues include noise associated with turbid water and difficultly identifying the river bed under water with a high concentration of sediments, debris, or gas bubbles and vegetation [24,25,26,27,28,29]. These techniques offer many advantages over carrying out underwater visual inspections, including improved health and safety of the inspectors, and are generally good in locations with poor access and poor visibility. A more comprehensive overview of scour monitoring devices can be found in studies by Kirby et al. [2], Kitchen et al. [10], Hunt [12], Fisher et al. [22], and Chen et al. [30]. 

A common drawback of conventional scour monitoring systems is that they measure the development of scour on the riverbed and not the effect of the scour on the stiffness and capacity of the structure. Direct monitoring of the changes in the structural behaviour of the bridge to discern the presence of scour has been taken forward by researchers in recent years, mainly in the field of modal analysis.

Xiong et al. [31], Xiong et al. [32], and Xiong et al. [33] carried out modal analyses of a bridge in the field before and after scouring took place, identifying the presence of scour by examining the support boundary conditions, i.e., the bridge foundations. The finite element (FE) model of the bridge was able to identify the depth of scour, as verified by an underwater terrain map of the area. Chen et al. [34] constructed an FE model, verified against a bridge in the field, and used it to perform a modal analysis. The best boundary support conditions were selected with a known soil depth at one of the piers, with the model then able to identify the scour depth at other piers as verified by onsite measurements. The effect of air temperature, water level, and traffic load on the results was examined by Wu et al. [35], with the traffic load being the most dominant of these factors (it is a long cable-stayed bridge with multiple traffic lanes). The effect of variation in temperature on the frequency is not as significant as variation in traffic loading. Also, it has been observed that the modal frequency baselines of the pier modes are very likely to increase with the increasing water level during the rainy season, but are constant during the summer when there is little change in the water level. Ju [36] also used an FE model validated against a bridge in the field to link bridge scour to a decrease in the natural frequency of the bridge. It was observed that the soil–fluid–structure interactions lower the natural frequency of the bridge, an important consideration if the bridge is submerged in water.

Kong and Cai [37] utilised an FE model approach to study several dynamic parameters of the substructure and superstructure, as well as a traversing vehicle, under the excitation of wave forces to detect scour damage. The findings include that scour has a relatively significant effect on the lower frequencies of a group of piles, and the frequency change of the foundation caused by the scour can also be detected from the response of the bridge deck as well as from the acceleration response from a vehicle moving on the bridge. Foti and Sabia [38] observed that the span of a bridge in the field which was sitting on a scoured pier had a lower fundamental frequency and an increased deformation of the span, and that a modal analysis proved to be less successful at identifying the scour. This is attributed to the rigid body motion of the piers.

Kariyawasam et al. [39] carried out a modal analysis in the laboratory on various bridge models and observed a decrease in the fundamental natural frequencies of the bridge models in response to scour. Khan et al. [40] and Khan et al. [41] performed a modal analysis using re-deployable accelerometers on a bridge in the laboratory, which was then tested on a bridge in the field. Prendergast and Gavin [42] and Prendergast et al. [43] carried out a full-scale field test on a pile to valid their numerical model, which estimates scour depth based on the observed frequency response of the pile. Bao et al. [44] examined the effect of asymmetric scour holes using a modal analysis of tests carried out in the laboratory. Fitzgerald et al. [45] used a cantilever-based piezoelectric energy harvesting device to detect a change in bridge frequency due to scour. Scozzese et al. [46] proposed a mode shape-oriented identification system for detecting scour, but concluded that noise compromises the results. OBrien et al. [47] focused on wavelet-derived operating deflection shapes from average bridge accelerations at supports, enabling the detection of scour over a range of frequencies, providing results in both the frequency and spatial domains.

As well as natural frequencies, Elsaid and Seracino [48] examined changes in mode shape curvature and flexibility-based deflections due to scour, but report that, due to the nature of the experiment, the results are inconclusive. Xiong et al. [49] and Xiong [50] discussed flexibility-based deflections, mode shape curvature, frequency change ratios, and modal assurance criteria. The frequency change ratio was only effective as a potential damage indicator on single-pier bridges, and the modal assurance criteria were found to be incapable of identifying scour. Flexibility-based deflections and mode shape curvature identify the modelled presence of scour. Catbas et al. [51] conducted a laboratory experiment where modal flexibility-based deflections and curvatures were calculated from modal frequencies and scaled mode shapes. The modelled scour was identified by an increase in the deflected shape patterns and modal curvature of the girders. Malekjafarian et al. [52] used the ratio between mode shape amplitudes identified at two points on an integral bridge structure to detect scour, and Malekjafarian et al. [53] evaluated a monitoring strategy based on the relative changes in pier-mode shape amplitudes based on a laboratory study. 

When considering a Structural Health Monitoring plan for an individual bridge, the cost of installation and difficulty of maintaining equipment need to be offset against the benefit of planning timely, efficient, and economical repairs and having information on the bridge’s likely remaining life [54,55]. 

There has been a growing interest in indirect Structural Health Monitoring, where a passing instrumented vehicle excites the bridge and the dynamic properties of the bridge are then extracted from the dynamic response of the vehicle. These techniques include the following:

– The detection of a change in the bridge’s natural frequency [56,57,58,59];
– Damping [60,61,62];
– The identification of mode shapes and modal strain energy [63,64,65,66,67];
– Wavelet transform techniques [68,69];
– Using moving force identification (MFI) to calculate the expected load if the bridge is in a heathy state [70,71];
– Using Machine Learning and Artificial Neural Networks (ANNs) to predict the ‘healthy’ response of the bridge [72,73];
– Indirectly measuring the relative displacement of the bridge [74,75,76,77,78].

Reviews of various indirect, or ‘drive-by’, bridge monitoring techniques are carried out in articles by Malekjafarian et al. [79], Shokravi et al. [80], and Yang and Yang [81]. There are few indirect monitoring techniques that specifically consider scour. Fitzgerald et al. [82] numerically investigated the feasibility of using bogie acceleration measurements from a passing train to detect the presence of bridge scour. The Continuous Wavelet Transform is used to process the simulated acceleration measurements for a number of train passages over the scoured bridge. 

This study examines the novel concept of measuring the rotation of a vehicle body, the ‘pitch’, as it crosses a bridge, to detect a change in bridge characteristics as a result of scour. The loss of stiffness caused by the erosion of the riverbed around the pier foundations will result in a change in the movement of the pier and the bridge deck under vehicular loading. The pitch of the crossing vehicle is in response to the ‘apparent profile’ of the bridge it experiences, which consists of two components: the road or rail profile and the bridge deflections in response to the crossing vehicle. The latter deflections are affected by the bridge boundary conditions and are damage-sensitive. A two-dimensional vehicle–bridge interaction FE model is utilised here to explore this concept, followed by laboratory experiments on a scaled 5.2 m long multi-span bridge consisting of a series of four 1.3 m simply supported spans.

As the sensors are mounted on a crossing vehicle, this technique becomes very economically beneficial as it has the ability to monitor many bridges on the network, including those that it may not be economically viable to undertake direct electronic Structural Health Monitoring. This is particularly important after a large-scale flood event which may result in many bridges being immediately reclassified as a high scour risk, and many monitoring techniques, especially traditional underwater inspections, also become a high health and safety risk. This scour monitoring technique also provides information on changes to the bridge’s structural behaviour, offering a better understanding of the bridge condition than isolated information on the location and depth of scouring.

## 2. Numerical Modelling

### 2.1. Description of Numerical Model

**Figure 1:**
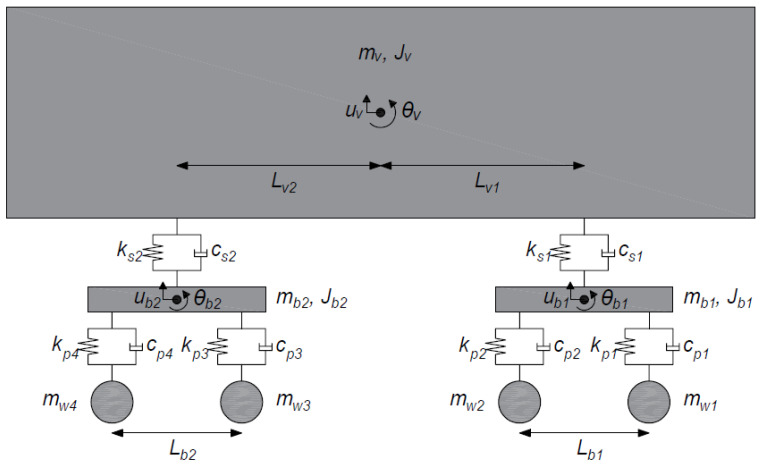
Schematic of vehicle model.

The vehicle is modelled as a series of three identical train carriages. A schematic of the train carriage is illustrated in Figure 1. Each carriage consists of a rigid vehicle body and two rigid bogies, each with a translation and rotation degree of freedom (DOF). Each of the four axles is associated with a translation DOF and each of the suspension systems is represented by a spring–dashpot unit. Each carriage is 24.56 m in length, with a distance of 2.56 m between axles on the same bogie and a distance of 16.44 m between the second and the third axles. The distance between the last axle of a carriage and the first axle of the next carriage is 6.00 m. The vehicle properties are listed in Table 1 and are based on those published by Iwnicki [83].

The multi-span bridge is modelled as four simply supported Euler–Bernoulli beams, each 12 m in length and consisting of 12 internal elements. A nodal hinge represents the connection between adjacent spans, allowing the beams to rotate separately but to share the same vertical translation. These rest on piers, each with an associated mass and stiffness, and consisting of a single DOF in the vertical direction. The vertical stiffness provided by a shallow pad foundation is modelled as a spring under each pier, values of which were obtained based on those of Adhikary et al. [84] and adjusted as proposed by Micu et al. [85]. The start and the end of the bridge rest on undeformable abutments (on the assumption that these are not in the water). There is a 25 m approach length, which is included to negate transient vehicle effects, and an exit length to allow the vehicle to clear the bridge. Using the Rayleigh damping approach, 3% damping is applied to the bridge spans–piers–foundation system [86].

**Figure 2:**
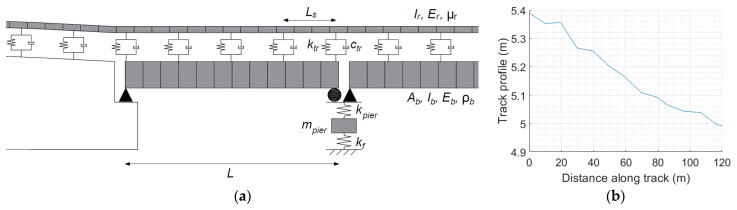
Numerical model of bridge: (**a**) bridge schematic showing piers, bridge deck, Winkler springs and track, and abutments; (**b**) longitudinal profile of rail track.

The rail track on the bridge is modelled as an Euler–Bernoulli beam resting on spring–dashpot units at 0.6 m intervals—see Figure 2a. Typical values were proposed by Zhai et al. [87] and are adopted here. An overview of these properties is presented in Table 2. A track profile is incorporated into the system based on an actual track profile survey taken on a bridge published by Micu et al. [85]—see Figure 2b.

The equations of the motion of the vehicle–bridge interaction system are coupled and time-dependent [88,89]. The numerical model is based on one detailed by Fitzgerald et al. [82] with further details by Fitzgerald [90].

With a specialised instrumented train, it is assumed that the majority of the train properties will remain unchanged between runs, with the exception of mass (which will be influenced by the mass of remaining fuel, persons onboard, etc.) and velocity (which can vary due to driver behaviour). To account for this natural variation, five populations, each consisting of ten vehicle runs with carriage body mass (i.e., excluding the bogie and wheelset mass) and velocity, are randomly selected from a normal distribution with a mean of 30,238 kg and 95 km/h, respectively, and a standard deviation of 5%. The percentage change from the default carriage body mass for each vehicle run is presented in Table 3, and the percentage change from the default velocity for each vehicle run is presented in Table 4.

In a ‘pad’ foundation, i.e., one without piles, the foundation pad sits directly on the soil. Scour has the effect of reducing the area of consolidated soil under the pad which reduces the effective stiffness as imposed loading passes overhead. Scour damage scenarios are modelled by reducing the stiffness of the foundation spring (representing the stiffness of the soil) under Pier 3, and then separately under Pier 2, each by 25% and then 50%, with the latter representing realistic extreme scour cases [46,91]. For each damage scenario, including the healthy scenario, a different population of vehicle runs is used.

### 2.2. Numerical Modelling Results

**Figure 3:**
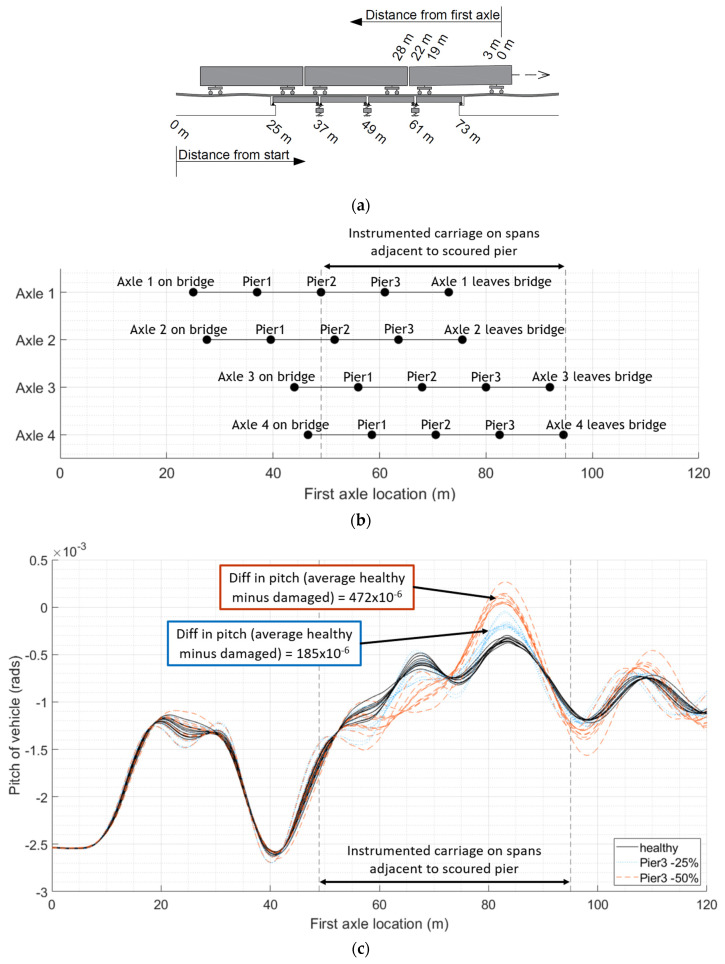
Comparing pitch of first carriage crossing bridge: (**a**) relative distances of span lengths from the start of the numerical model and relative distances of axle spacings from the first axle; (**b**) position of each axle of the first train carriage relative to the location of the first axle and the location of the bridge piers; (**c**) pitch of vehicles with varying mass and velocity crossing bridge with increasing loss of stiffness under Pier 3.

Figure 3 illustrates the rotation of the carriage body of the first carriage of the train as it travels across the 25 m approach length to the bridge, and then over the four 12 m long bridge spans. This is repeated for populations of train runs, both for the healthy bridge and when there is scour under Pier 3. Figure 3a gives the distance of each axle along the train, and the distance of each span from the start of the model. Figure 3b shows the progression of each axle of the first carriage across the bridge relative to the location of the first axle. Figure 3c shows the rotation of the carriage body of the first carriage of the train as it crosses the bridge. The pitch of the vehicle is in response to the apparent profile it experiences, a combination of the vertical profile of the track and the deflections of the bridge in response to the vehicle. A loss of foundation stiffness under a particular pier will influence the apparent profile of the adjacent spans. For scour under Pier 3, the first axle will reach the first adjacent span after travelling 49 m (i.e., 25 m approach length + 2 × 12 m spans) and will clear both adjacent spans after travelling 73 m (i.e., 25 m approach length + 4 × 12 m spans). The last axle of the first carriage will reach the spans adjacent to the scoured pier 22 m later, i.e., when the first axle is at 71 m, and will clear them when the first axle reaches 95 m. It can be seen in Figure 3c that, over this carriage interaction distance (from 49 m to 95 m), there is a clear divergence in the pitch for each population of vehicles, both from the healthy scenario when scour is present. The mean difference in the maximum pitch from a healthy baseline (as shown with solid black plots) and the pitch as a result of a 25% loss of stiffness (as shown with dotted blue plots) is 185 × 10^−6^ radians, and the mean difference in maximum pitch between the healthy baseline and a 50% loss of stiffness (as shown with dashed red plots) is 472 × 10^−6^ radians.

**Figure 4:**
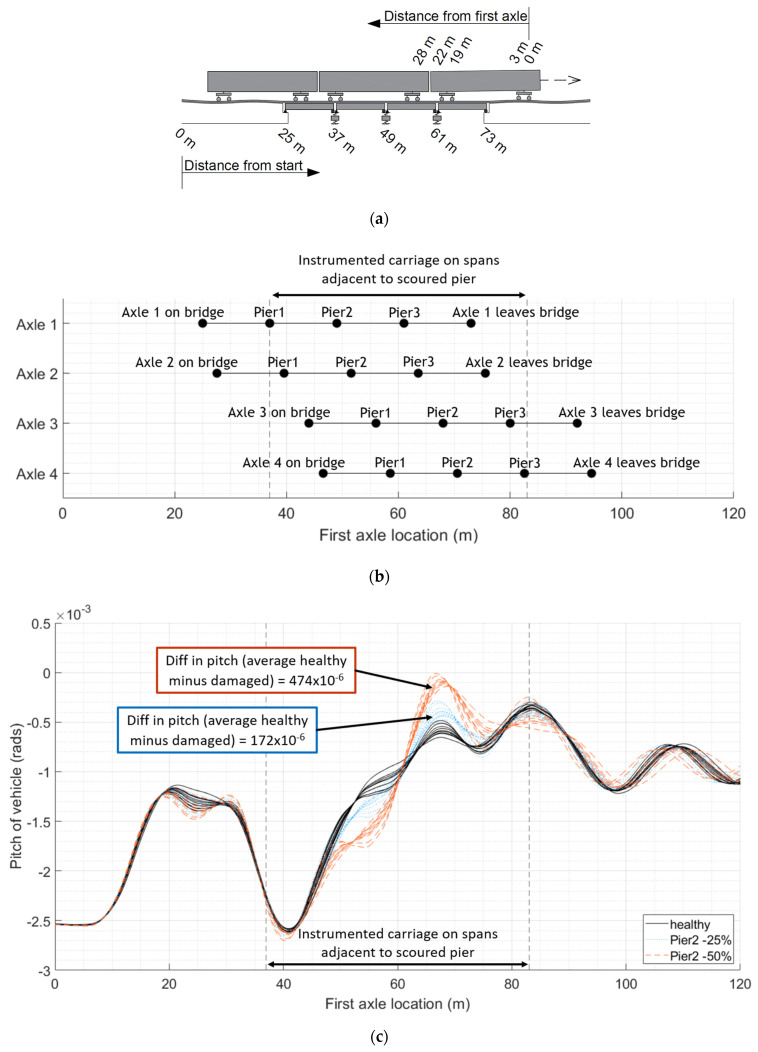
Comparing pitch of first carriage crossing bridge: (**a**) relative distances of span lengths from the start of the numerical model and relative distances of axle spacings from the first axle; (**b**) position of each axle of the first train carriage relative to the location of the first axle and the location of the bridge piers; (**c**) pitch of vehicles with varying mass and velocity crossing bridge with increasing loss of stiffness under Pier 2.

We can see a similar trend in Figure 4, which gives the first carriage pitch for the healthy baseline case (as shown with solid black plots), for 25% stiffness loss under Pier 2 (as shown with dotted blue plots) and for 50% stiffness loss under Pier 2 (as shown with dashed red plots). The first axle of the carriage will reach the adjacent spans to the scoured pier after travelling 37 m and clear the spans at 61 m, with the last axle of the carriage reaching the adjacent spans when the first axle is at 59 m and clears them when the first axle is at 83 m. The mean difference in the maximum pitch between the scoured scenarios and the healthy baseline is 172 × 10^−6^ radians for 25% loss and 474 × 10^−6^ radians for 50% loss.

## 3. Laboratory Testing

### 3.1. Experimental Setup

**Figure 5:**
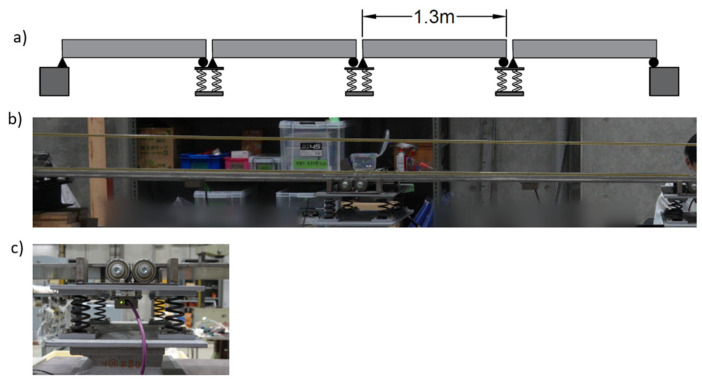
Test structure. (**a**) Elevation sketch; (**b**) partial elevation view showing internal pier and adjacent spans; (**c**) roller end support on pier.

The laboratory test structure is a multi-span bridge consisting of four 1.3 m long simply supported spans supported on three internal piers on springs and two rigid abutments—see Figure 5. The cross-section is a 300 mm wide, 8.07 mm deep steel plate with two 8 mm square steel rails, which provide a track for a test vehicle to drive on. The rail profile is level. The model properties, which were supplied by the manufacturer, are given in Table 5.

Each pier is founded on a base of four springs, each of stiffness 49 × 10^3^ N/m, providing a total stiffness under each pier of 196 × 10^3^ N/m. The scale of the test structure is defined by the ratio of the deflection of the midspan under a unit load at that point and the deflection of the pier under a unit load at the pier. This ratio is the same for the scaled test structure as it would be for an equivalent full-scale bridge.

The midspan deflection due to a unit load at midspan is

(1) $$\delta_{midspan}=\frac{L^{3}}{48EI}$$

where *L* = span length;

*E* = Young’s Modulus;

*I* = second moment of area.

The pier deflection due to a unit load at that point is

(2) $$\delta_{pier}=\frac{1}{k}$$

where *k* = the equivalent stiffness of pier springs.

Taking the ratio of the deflection of the midspan under a unit load at the midspan (Equation (1)) to the deflection of the pier under a unit load at the pier (Equation (2)) and equating this ratio for the experimental test case and an actual full-scale bridge gives the equivalent scaled stiffness under the pier of the test structure:

(3) $$k_{exp}=k_{act}\left(\frac{L_{act}^{3}\;E_{exp}\;I_{exp}}{L_{exp}^{3}\;E_{act}\;I_{act}}\right)$$

where *_exp_* = experimental test case;

*_act_* = actual full-scale bridge.

Using Equation (3), the stiffness under a pier in an equivalent full-scale bridge can be calculated as 2.34 × 10^6^ N/m. A shallow pad foundation of length 4 m and width 2 m is assumed. As per Adhikary et al. [84] and Prendergast and Gavin [92], a stiffness of 1.72 × 10^6^ N/m can be expected for loose sand, with 3.44 × 10^6^ N/m for medium-dense sand. The calculated stiffness provided by the springs under each pier in the test structure lies between these two and is assumed to be the equivalent of a founding on loose-to-medium-dense uniform sand in a comparable full-scale bridge. The springs under individual piers are replaced with springs of lower stiffness to replicate the effects of scouring undermining the shallow foundations and leading to settlement [93]. As for the numerical models, the damage scenarios explored in this study are a 25% and 50% loss of stiffness under Piers 2 and 3 separately.

**Figure 6:**
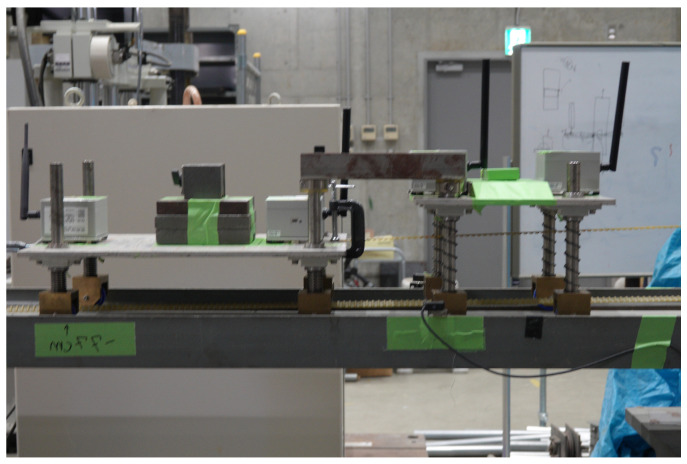
Test vehicle (showing tractor–trailer left to right).

The vehicle used is the four-axle tractor–trailer shown in Figure 6, with the vehicle properties listed in Table 6. It travels across the test structure at a constant velocity of 1.14 m/s, controlled electronically by a pulley system, with acceleration and deceleration spans on either side of the test structure. The test vehicle carries wireless Epson M-A352 triaxial accelerometers (on both the body of the tractor and trailer) and data acquisition was at a 200 Hz sampling rate. Additional mass is added to the vehicle by securing weights to the vehicle body.

### 3.2. Data Processing

**Figure 7:**
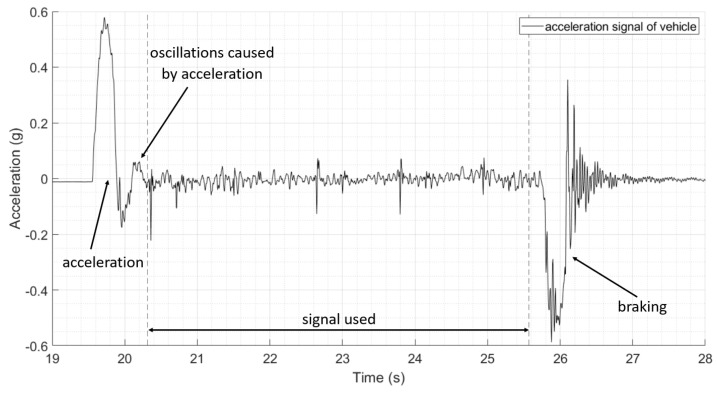
Typical acceleration signal from one vehicle crossing bridge in the time domain.

A typical raw acceleration signal (from the axis aligned with the direction of travel) is shown in Figure 7. The sudden acceleration and deceleration of the vehicle results in large peaks at the beginning and end of the signal. These occur on the acceleration and deceleration ramps at either end of the bridge, with the resulting oscillations caused by the acceleration continuing into the beginning of the bridge before damping out. These peaks and associated oscillations dominate the acceleration signal and are removed before the signal is filtered to remove the high-frequency content. This results in a small portion of the signal, which corresponds to when the vehicle is on the start of the first span of the bridge, to also be removed. The portion of the signal used is indicated in Figure 7. The remaining signal is filtered using a sixth-order low-pass digital Butterworth filter with a normalised cut-off frequency of 1 Hz. The resulting filtered signal will consist of the static component of the signal.

The accelerometers used on the test vehicle are sensitive enough to detect frequencies as low as 0 Hz (i.e., gravity), and hence can be used to convert the acceleration signal into rotation. The relationship between the static component of an accelerometer reading and its angle of rotation is sinusoidal. When an accelerometer is orientated vertically, it records ±1 g, or ±9.81 m/s^2^, and when orientated horizontally, it records zero. Using simple trigonometry, the output signal is converted into rotation as per Equation (4).

(4) $$\Theta ={sin}^{-1}\left(\frac{a}{g}\right)$$

where *a* = accelerometer reading;

*g* = acceleration due to gravity (i.e., 9.81 m/s^2^);

Θ = angle of rotation.

**Figure 8:**
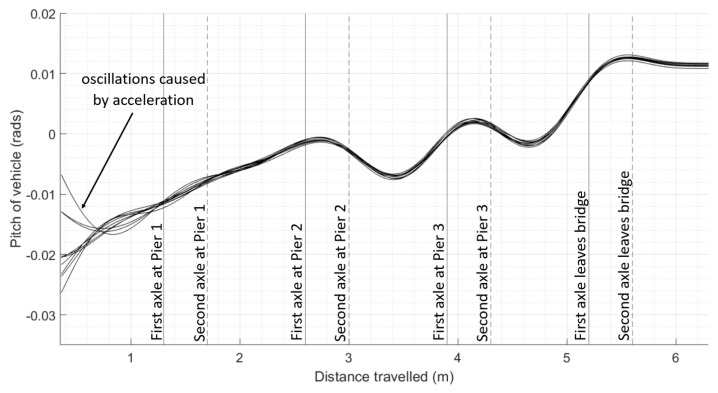
Pitch of vehicle in 10 different journeys as it travels along the healthy bridge, affected by sudden acceleration at the start of each journey.

Each test population consists of 10 vehicle runs across the bridge. Rotations from the 10 healthy bridge journeys are illustrated in Figure 8. The graph shows the measured pitch of the vehicle tractor (which can rotate independently of the trailer) as it travels across the bridge. The location of the vehicle on the bridge is given relative to when the first axle crosses onto the first span. The test bridge itself does not sit level, with the underlying pitch of the vehicle increasing from 4.7 × 10^−3^ radians to 11.9 × 10^−3^ radians (a gradual increase in the region of 0.4°).

Good repeatability can be seen in the values obtained, apart from the first metre of the journey, which is affected by the sudden acceleration experienced by the vehicle. It is recommended that, in future experimental setups, a longer approach span is used. It can also be seen that the spans before and after Pier 1 do not deflect under the weight of the vehicle as expected. This may be due to some unanticipated restraint in rotation, as, during the experiment, there was longitudinal movement of the first span and contact between the spans at Pier 1. From Pier 2 onwards, we can see the expected structural behaviour of the simply supported spans (with a level road profile), with the vehicle experiencing a negative pitch as it travels towards the increasing deflecting midspan of the beams, and a positive pitch as the vehicle passes the midspan and the deflection of the beam starts to reduce.

### 3.3. Results

**Figure 9:**
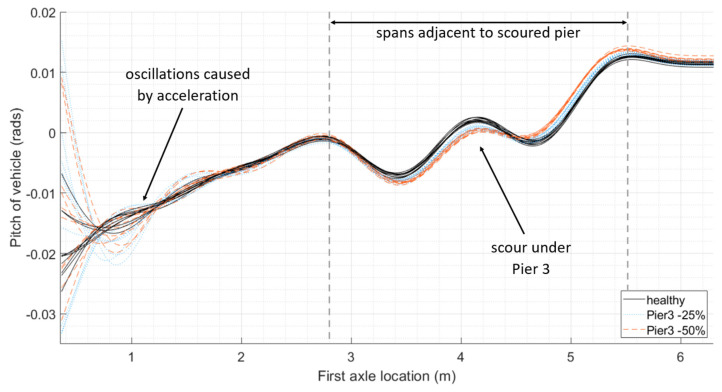
Pitch of vehicle in 10 different journeys for each damage scenario as it travels along the bridge with increasing levels of damage under Pier 3.

The effect of the modelled scour under Pier 3 on vehicle pitch is shown in Figure 9, where the healthy test scenario (as shown with solid black plots) is compared to a 25% (as shown with dotted blue plots) and 50% (as shown with dashed red plots) reductions in stiffness under the pier. No discernible difference can be observed between the pitch of the vehicle on the three different test scenarios until the vehicle is on the spans adjacent to the scoured pier. On the approach to the scoured pier, the increase in vehicle pitch lessens for both scour conditions, and once past the pier, the decrease in vehicle pitch is also lessened for both scour conditions. This is apparent while the vehicle is on either of the spans adjacent to the scoured pier. The maximum difference of the mean vehicle pitch between the healthy runs and the 25% damage runs is 1.5 × 10^−3^ radians in the vicinity of Pier 3, and the difference in the mean vehicle pitch between the healthy runs and the 25% damage runs is 2.0 × 10^−3^ radians.

**Figure 10:**
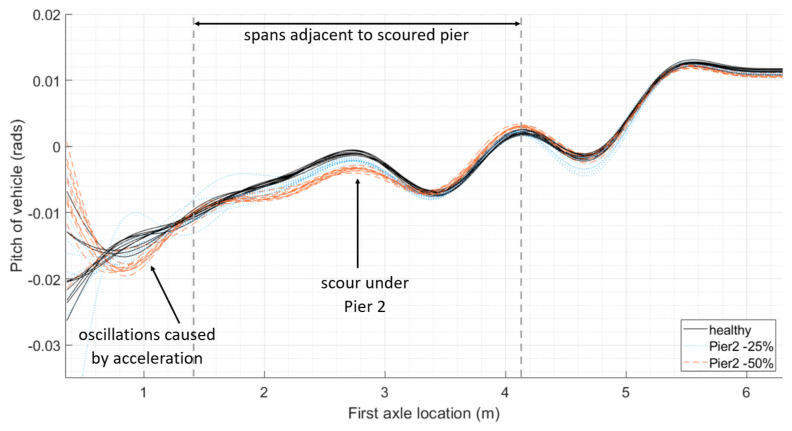
Pitch of vehicle in 10 different journeys for each damage scenario as it travels along the bridge with increasing levels of damage under Pier 2.

Scour is modelled as a 25% loss of stiffness (as shown with dotted blue plots) and a 50% loss of stiffness (as shown with dashed red plots) under Pier 2 in Figure 10. Again, we can see the effect this has on vehicle pitch, with the maximum difference of the mean vehicle pitch between the healthy runs and the 25% damage runs being 1.3 × 10^−3^ radians in the vicinity of Pier 2, and the difference in the mean vehicle pitch between the healthy runs and the 25% damage runs is 2.7 × 10^−3^ radians.

**Figure 11:**
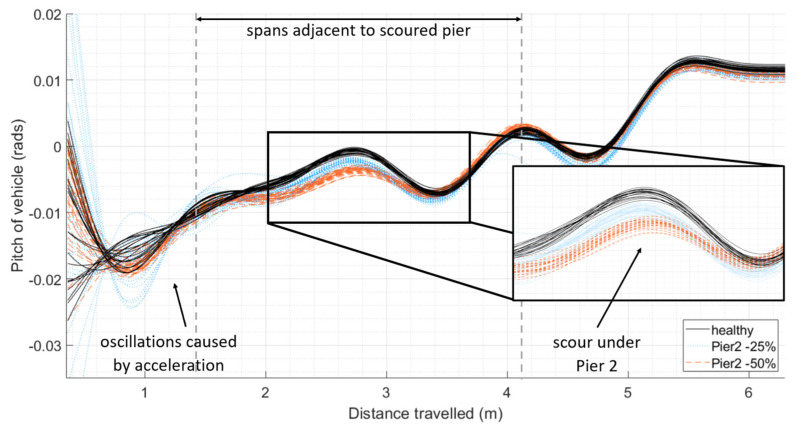
Pitch of vehicle in 30 different journeys for each damage scenario as it travels along the bridge with increasing levels of damage under Pier 2: 10 journeys with −5% mass per damage scenario, 10 journeys with the default mass per damage scenario, and 10 journeys with +5% mass per damage scenario.

The previous scenario, which models scour under Pier 2, as depicted in Figure 10, is repeated with an additional 10 runs of the vehicle with an increased mass of 5%, and a further 10 runs with the mass of the vehicle reduced by 5% for the healthy scenario (30 solid black plots), a 25% loss of stiffness (30 dotted blue plots), and a 50% loss of stiffness (30 dashed red plots). This is shown in Figure 11, where it can be seen that there continues to be a clear separation between the healthy and damaged scenarios.

## 4. Further Research

This study provides the concept and preliminary validation of a new bridge condition monitoring methodology. The initial proof of concept is described in this paper. To extend this work, the authors would like to develop the laboratory testing further. Scour damage was simulated as a reduction in spring stiffness under piers of a multi-span bridge. This could be expanded in the future and modelled in the laboratory using a box of soil to support the piers, with the material being removed in a way to mimic different scouring scenarios, including lesser levels of damage. The authors would see the benefit of modelling different types of bridges and foundations, along with a different vehicle pulley system capable of travelling at higher velocities. The oscillatory nature of the measured response can be amplified or dampened by the presence of damage, particularly when combined with a track profile, as demonstrated by the numerical modelling at the start of this study (Figure 2, Figure 3 and Figure 4). A track profile was not included in the laboratory tests to better understand the bridge behaviour. Future modelling will include different track profiles.

Bridge structures generally exhibit bending or arch behaviour. For both, if the foundation settles under a passing load, the effect on the carriage rotation is similar. Although the in-span behaviour may differ for different bridge types, the methodology can be extended to different types of bridges. Bridges with longer spans will have a reduced slope for a given settlement resulting from scour, which will make it harder to detect the deflection. This will be more of a challenge and will require more passes of the instrumented vehicle.

A 3D model will be developed in future research, mimicking likely bridges to be monitored. This will allow for more comprehensive and varied damage modelling to be undertaken and help to develop a regression formula for scour detection under set circumstances. With the aim of monitoring the deterioration of bridges, the methodology can be scaled up for medium-span bridges, which typically would not be bespoke structures, but are often precast and can be grouped together into those with similar designs. 

The sensors used in this research are wireless Epson M-A352 triaxial accelerometers (Epson, Suwa, Japan), measuring data along three axes. This allows the rotation of the vehicle up and down to be measured, i.e., the vehicle pitch. It also measures the circular (clockwise or anticlockwise) movement of the vehicle as it moves forward, i.e., the vehicle roll. This may be an effective way of measuring potential movement of the bridge pier in the lateral direction if the pattern of scour results in one side of a pier settling.

Although the proposed methodology ultimately does not require a digital twin, which is resource-intensive to scale-up for a large quantity of bridges, it instead utilises a comparison to a healthy baseline. A disadvantage of this approach is adjusting for routine behavioural changes in the structure, for example, as a result of temperature fluctuations throughout the year. Techniques to deal with variations in parameters, such as temperature, can involve grouping together vehicle runs with similar parameters for comparison, resulting in several ‘healthy baselines’ [85].

Scaling this methodology up for monitoring real-world bridges would require the installation of sensors on the vehicles. The sensors are installed on the carriage body of the train, which affords more protection and ease of access than installation on the bogie or axle. Within this study, slight variations in mass and the velocity of the train are included. This would be more reflective of a measurement train and not a passenger carriage, which would likely experience greater fluctuations in mass. Variations in parameters, including changes to the suspension of the vehicle over time, would need to be explored further for long-term monitoring.

## 5. Conclusions

This study proposes a new damage monitoring concept based on the comparison of the pitch of a passing train carriage between a healthy and a scoured bridge. The pitch of a crossing vehicle changes in response to the apparent profile as it crosses the structure. Further, the pattern of the pitch signal of the carriage body is damage-sensitive and changes as a result of a loss of stiffness under a pier.

The concept was initially trialled on a numerical study: a two-dimensional vehicle–bridge interaction model consisting of a three-carriage train travelling along a rail track overlaying a bridge with four simply supported spans. Scour was modelled as a loss of stiffness under a bridge pier. Sensitivity testing included adjustments for carriage mass and velocity. The results showed a clear and distinctive change in vehicle pitch as a result of increasing levels of scour when compared to the healthy bridge.

The concept was also tested in a scaled laboratory experiment, where a tractor–trailer repeatedly crossed a 5.2 m long bridge supported on two end abutments and three springs representing piers. Scour was applied to the model by exchanging the springs under a ‘scoured’ pier with those of a lesser stiffness. While there were considerable challenges, the results show a clear relationship between vehicle pitch and loss of stiffness due to scouring.

## Figures and Tables

**Table 1:** Numerical model vehicle properties.

Property	Symbol	Unit	Value
Carriage body mass	*m_v_*	kg	30,238
Carriage body moment of inertia	*J_v_*	kg m^2^	2.119 × 10^6^
Bogie mass	*m_b_*	kg	2615
Bogie moment of inertia	*J_b_*	kg m^2^	1476
Wheelset mass	*m_w_*	kg	1813
Primary suspension stiffness	*k_p_*	N m^−1^	2.40 × 10^6^
Secondary suspension stiffness	*k_s_*	N m^−1^	0.86 × 10^6^
Primary suspension damping	*c_p_*	kN s m^−1^	7
Secondary suspension damping	*c_s_*	kN s m^−1^	16
Distance between axles	*L_bi_*	m	2.56
Horizontal distance between centre of mass of main body and bogie	*L_v_*	m	9.50

**Table 2:** Numerical model bridge properties.

Property	Symbol	Unit	Value
Span length	*L*	mm	12,000
Beam cross-sectional area	*A_b_*	mm^2^	4 × 10^6^
Beam second moment of area	*I_b_*	mm^4^	83.3 × 10^9^
Beam modulus of elasticity	*E_b_*	N m^−2^	205 × 10^9^
Beam density	*ρ_b_*	kg m^−3^	9600
Underlying foundation stiffness	*k_f_*	N mm^−1^	0.3 × 10^9^
Pier mass	*m_pier_*	kg	42,000
Pier stiffness	k_pier_	N mm^−1^	12.5 × 10^12^
Rail Young’s modulus	*E_r_*	N m^−2^	206 × 10^9^
Rail second moment of area	*I_r_*	m^4^	32 × 10^−6^
Rail mass per unit length	*μ_r_*	kg m^−1^	60.64
Track stiffness	k*_tr_*	N m^−1^	138 × 10^6^
Track damping	c*_tr_*	kN s m^−1^	75 × 10^3^
Sleeper spacing	L*_s_*	m	0.6

**Table 3:** Variation from default carriage body mass of 30,238 kg within each population of vehicles.

	Population 1	Population 2	Population 3	Population 4	Population 5
Run 1	−11.3%	−6.7%	−6.0%	−14.7%	−4.3%
Run 2	−6.5%	−1.0%	−3.9%	−8.6%	−1.2%
Run 3	−2.2%	−0.6%	−1.5%	−5.7%	−0.8%
Run 4	1.6%	−0.3%	1.5%	−5.3%	−0.5%
Run 5	1.7%	3.6%	2.4%	−4.0%	−0.2%
Run 6	2.7%	3.6%	3.4%	−3.8%	1.6%
Run 7	4.3%	7.0%	3.6%	1.6%	1.6%
Run 8	9.2%	7.1%	3.6%	4.4%	3.1%
Run 9	13.8%	7.4%	5.2%	6.9%	5.5%
Run 10	17.9%	15.2%	8.2%	7.2%	5.5%

**Table 4:** Variation from default velocity of 95 km/h within each population of vehicles.

	Population 1	Population 2	Population 3	Population 4	Population 5
Run 1	−4.4%	−10.7%	−10.3%	−7.9%	−10.0%
Run 2	−3.0%	−9.8%	−5.3%	−6.7%	−8.8%
Run 3	−2.7%	−6.0%	−2.3%	−4.1%	−5.8%
Run 4	−1.0%	−5.4%	−1.4%	−1.8%	−4.9%
Run 5	0.5%	−4.2%	−1.4%	−1.5%	−4.2%
Run 6	1.5%	−1.0%	3.5%	0.2%	−2.7%
Run 7	2.4%	0.6%	4.1%	1.4%	−1.4%
Run 8	3.7%	4.8%	5.5%	1.8%	−1.3%
Run 9	4.2%	6.8%	6.9%	2.5%	0.1%
Run 10	8.6%	7.2%	14.5%	5.6%	4.8%

**Table 5:** Span details.

Span length	1.3 m
Beam width	0.3 m
Beam depth	8.07 × 10^−3^ m
Young’s modulus	205 × 10^9^ N m^−2^
Density	7.85 × 10^3^ kg m^−3^
Second moment of area	1.31 × 10^−6^ m^4^

**Table 6:** Test vehicle properties.

	Tractor		Trailer	
Weight (kg) (excluding axle weights)	22.0		11.6	
	Axle 1	Axle 2	Axle 3	Axle 4
Axle weights (kg)	0.9	0.9	0.9	0.9
Spring stiffness (N/m)	1522	1827	4000	4000
Distance from previous axle (mm)	-	400	220	200

## Data Availability

The raw data supporting the conclusions of this article will be made available by the authors on request.

## References

[1] Hamill L. (1999). Bridge Hydraulics. E. and F.N. Spon, London. Adv. Water Resour..

[2] Kirby A., Roca M., Kitchen A., Escarameia M., Chesterton J. (2015). Manual on Scour at Bridges and Other Hydraulic Structures.

[3] Wardhana K., Hadipriono F.C. (2003). Analysis of Recent Bridge Failures in the United States. J. Perform. Constr. Facil..

[4] Jongman B., Hochrainer-Stigler S., Feyen L., Aerts J.C.J.H., Mechler R., Botzen W.J.W., Bouwer L.M., Pflug G., Rojas R., Ward P.J. (2014). Increasing stress on disaster-risk finance due to large floods. Nat. Clim. Chang..

[5] Richard M., Martin H. (2019). Winter monitoring of damaged bridges: Learning from the December 2015 floods in Cumbria, UK. Proc. Inst. Civ. Eng. Eng. Sustain..

[6] Giordano P.F., Limongelli M.P., Prendergast L. Impact of climate change on the Value of Information for bridges at risk of scour. Proceedings of the 7th International Symposium on Life-Cycle Civil Engineering (IALCCE2020).

[7] RAIU (2010). Investigation Report No. 2010—R004: Malahide Viaduct Collapse on the Dublin to Belfast Line on the 21st August 2009.

[8] Antunes do Carmo J. (2021). River Basin Management—Sustainability Issues and Planning Strategies.

[9] Maddison B. (2012). Scour failure of bridges. Proc. Inst. Civ. Eng. Forensic Eng..

[10] Kitchen A., Roca M., Kirby A.M., Escarameia M. (2021). Manual on Scour at Bridges and Other Hydraulic Structures—Supplementary Guide.

[11] Briaud J.-L., Hurlebaus S., Chang K.-A., Yao C., Sharma H., Yu O.-Y., Darby C., Hunt B.E., Price G.R. (2011). Realtime Monitoring of Bridge Scour Using Remote Monitoring Technology (Report No: 0-6060-1).

[12] Hunt B. (2009). NCHRP Synthesis 396, Monitoring Scour Critical Bridges.

[13] Lagasse P.F., Richardson E.V., Schall J.D., Price G.R. (1997). Instrumentation for Measuring Scour at Bridge Piers and Abutments.

[14] Chen G., Tang Y., Chen Z., Guo C.R., Bao Y., Fan L., Hu X., Klegseth M., Li Z. (2016). Smart Rock Technology for Real-Time Monitoring of Bridge Scour and Riprap Effectiveness—Design Guidelines and Visualization Tool.

[15] Tang F., Chen Y., Guo C., Fan L., Chen G., Tang Y. (2019). Field Application of Magnet-Based Smart Rock for Bridge Scour Monitoring. J. Bridge Eng..

[16] Zhang H., Li Z., Chen G., Reven A., Scharfenberg B., Ou J. (2021). UAV-based smart rock localization for bridge scour monitoring. J. Civ. Struct. Health Monit..

[17] Kong X., Cai C., Hu J., Xiong W., Peng H. (2016). Field Application of an Innovative Bridge Scour Monitoring System with Fiber Bragg Grating Sensors. J. Aerosp. Eng..

[18] Lin Y.B., Lai J.S., Chang K.C., Li L.S. (2006). Flood scour monitoring system using fiber Bragg grating sensors. Smart Mater. Struct..

[19] Zarafshan A., Iranmanesh A., Ansari F. (2012). Vibration-Based Method and Sensor for Monitoring of Bridge Scour. J. Bridge Eng..

[20] Maroni A., Tubaldi E., Ferguson N., Tarantino A., McDonald H., Zonta D. (2020). Electromagnetic Sensors for Underwater Scour Monitoring. Sensors.

[21] Michalis P., Tarantino A., Tachtatzis C., Judd M.D. (2015). Wireless monitoring of scour and re-deposited sediment evolution at bridge foundations based on soil electromagnetic properties. Smart Mater. Struct..

[22] Fisher M., Chowdhury M.N., Khan A.A., Atamturktur S. (2013). An evaluation of scour measurement devices. Flow Meas. Instrum..

[23] Yu X., Yu X. (2009). Time Domain Reflectometry Automatic Bridge Scour Measurement System: Principles and Potentials. Struct. Health Monit..

[24] Anderson N.L., Ismael A.M., Thitimakorn T. (2007). Ground-penetrating radar: A tool for monitoring bridge scour. Environ. Eng. Geosci..

[25] Campbell K.E.J., Ruffell A., Pringle J., Hughes D., Taylor S., Devlin B. (2021). Bridge Foundation River Scour and Infill Characterisation Using Water-Penetrating Radar. Remote Sens..

[26] Clubley S., Manes C., Richards D. (2015). High-resolution sonars set to revolutionise bridge scour inspections. Proceedings of the Institution of Civil Engineers. Civ. Eng..

[27] De Falco F., Mele R. (2002). The monitoring of bridges for scour by sonar and sedimetri. NDT E Int. Indep. Nondestruct. Test. Eval..

[28] Hayden J.T., Puleo J.A. (2011). Near Real-Time Scour Monitoring System: Application to Indian River Inlet, Delaware. J. Hydraul. Eng..

[29] Selvakumaran S., Plank S., Geiß C., Rossi C., Middleton C. (2018). Remote monitoring to predict bridge scour failure using Interferometric Synthetic Aperture Radar (InSAR) stacking techniques. Int. J. Appl. Earth Obs. Geoinf..

[30] Chen W., Yu X., Fayun L. (2017). A review of bridge scour; mechanism, estimation, monitoring and countermeasures. Nat. Hazards.

[31] Xiong W., Cai C.S., Kong B., Zhang X., Tang P. (2019). Bridge Scour Identification and Field Application Based on Ambient Vibration Measurements of Superstructures. J. Mar. Sci. Eng..

[32] Xiong W., Wei L., Zhang X., Wang W., Ye J. (2019). Dynamic-based bridge scour identification of super-span cable-supported bridges. Harbin Gongye Daxue Xuebao J. Harbin Inst. Technol..

[33] Xiong W., Zhang X., Tang P., Wang B., Ye J. (2018). Scour condition analysis on pylons of Hangzhou Bay Bridge by tracing dynamic behaviors of superstructures. Dongnan Daxue Xuebao J. Southeast Univ..

[34] Chen C.-C., Wu W.-H., Shih F., Wang S.-W. (2014). Scour evaluation for foundation of a cable-stayed bridge based on ambient vibration measurements of superstructure. NDT E Int. Indep. Nondestruct. Test. Eval..

[35] Wu W.-H., Chen C.-C., Shi W.-S., Huang C.-M. Possible environmental effects in scour monitoring of a Cable-stayed Bridge with pier vibration measurements. Proceedings of the IEEE International Instrumentation and Measurement Technology Conference.

[36] Ju S.H. (2013). Determination of scoured bridge natural frequencies with soil–structure interaction. Soil Dyn. Earthq. Eng..

[37] Kong X., Cai C.S. (2016). Scour Effect on Bridge and Vehicle Responses under Bridge–Vehicle–Wave Interaction. J. Bridge Eng..

[38] Foti S., Sabia D. (2011). Influence of Foundation Scour on the Dynamic Response of an Existing Bridge. J. Bridge Eng..

[39] Kariyawasam K.D., Middleton C.R., Madabhushi G., Haigh S.K., Talbot J.P. (2020). Assessment of bridge natural frequency as an indicator of scour using centrifuge modelling. J. Civ. Struct. Health Monit..

[40] Khan M.A., McCrum D.P., Prendergast L.J., OBrien E.J., Fitzgerald P.C., Kim C.-W. (2021). Laboratory investigation of a bridge scour monitoring method using decentralized modal analysis. Struct. Health Monit..

[41] Khan M.A., McCrum D.P., OBrien E.J., Bowe C., Hester D., McGetrick P.J., O’Higgins C., Casero M., Pakrashi V. (2022). Re-deployable sensors for modal estimates of bridges and detection of damage-induced changes in boundary conditions. Struct. Infrastruct. Eng..

[42] Prendergast L.J., Gavin K. Monitoring of scour critical bridges using changes in the natural frequency of vibration of foundation piles—A field investigation. Proceedings of the Transport Research Arena 5th Conference: Transport Solutions from Research to Deployment.

[43] Prendergast L.J., Hester D., Gavin K., O’Sullivan J.J. (2013). An investigation of the changes in the natural frequency of a pile affected by scour. J. Sound Vib..

[44] Bao T., Swartz A.R., Vitton S., Sun Y., Zhang C., Liu Z. (2017). Critical insights for advanced bridge scour detection using the natural frequency. J. Sound Vib..

[45] Fitzgerald P.C., Malekjafarian A., Bhowmik B., Prendergast L.J., Cahill P., Kim C., Hazra B., Pakrashi V., OBrien E.J. (2019). Scour Damage Detection and Structural Health Monitoring of a Laboratory-Scaled Bridge Using a Vibration Energy Harvesting Device. Sensors.

[46] Scozzese F., Ragni L., Tubaldi E., Gara F. (2019). Modal properties variation and collapse assessment of masonry arch bridges under scour action. Eng. Struct..

[47] OBrien E.J., McCrum D.P., Khan M.A., Prendergast L.J. (2023). Wavelet-based operating deflection shapes for locating scour-related stiffness losses in multi-span bridges. Struct. Infrastruct. Eng..

[48] Elsaid A., Seracino R. (2014). Rapid assessment of foundation scour using the dynamic features of bridge superstructure. Constr. Build. Mater..

[49] Xiong W., Cai C.S., Kong B., Tang P., Ye J. (2018). Identification of Bridge Scour Depth by Tracing Dynamic Behaviors of Superstructures. KSCE J. Civ. Eng..

[50] Xiong W., Kong B., Tang P., Ye J. (2018). Vibration-Based Identification for the Presence of Scouring of Cable-Stayed Bridges. J. Aerosp. Eng..

[51] Catbas F.N., Gul M., Burkett J.L. (2008). Conceptual damage-sensitive features for structural health monitoring: Laboratory and field demonstrations. Mech. Syst. Signal Process..

[52] Malekjafarian A., Prendergast L.J., O Brien E. (2020). Use of mode shape ratios for pier scour monitoring in two-span integral bridges under changing environmental conditions. Can. J. Civ. Eng..

[53] Malekjafarian A., Kim C.-W., O Brien E., Prendergast L., Fitzgerald P., Nakajima S. (2020). Experimental Demonstration of a Mode Shape-Based Scour-Monitoring Method for Multispan Bridges with Shallow Foundations. J. Bridge Eng..

[54] Giordano P.F., Prendergast L.J., Limongelli M.P. (2020). A framework for assessing the value of information for health monitoring of scoured bridges. J. Civ. Struct. Health Monit..

[55] Lydon M., Taylor S., Robinson D., Mufti A., OBrien E. (2015). Recent developments in bridge weigh in motion (B-WIM). J. Civ. Struct. Health Monit..

[56] Lin C.W., Yang Y.B. (2005). Use of a passing vehicle to scan the fundamental bridge frequencies: An experimental verification. Eng. Struct..

[57] Siringoringo D.M., Fujino Y. (2012). Estimating Bridge Fundamental Frequency from Vibration Response of Instrumented Passing Vehicle: Analytical and Experimental Study. Adv. Struct. Eng..

[58] Toshinami T., Kawatani M., Kim C.-W. Feasibility investigation for identifying bridge’s fundamental frequencies from vehicle vibrations. Proceedings of the 5th International Conference of Bridge Maintenance, Safety and Management (IABMAS2010).

[59] Yang Y.B., Lin C.W. (2005). Vehicle–bridge interaction dynamics and potential applications. J. Sound Vib..

[60] González A., OBrien E.J., McGetrick P.J. (2012). Identification of damping in a bridge using a moving instrumented vehicle. J. Sound Vib..

[61] He W.-Y., He J., Ren W.-X. (2018). Damage localization of beam structures using mode shape extracted from moving vehicle response. Meas. J. Int. Meas. Confed..

[62] McGetrick P.J., González A., OBrien E.J. (2009). Theoretical investigation of the use of a moving vehicle to identify bridge dynamic parameters. Insight.

[63] Kong X., Cai C.S., Deng L., Zhang W. (2017). Using Dynamic Responses of Moving Vehicles to Extract Bridge Modal Properties of a Field Bridge. J. Bridge Eng..

[64] Locke W., Redmond L., Schmid M. (2021). Experimental Evaluation of Drive-by Health Monitoring on a Short Span Bridge Using OMA Techniques. Dynamics of Civil Structures, Volume 2: Proceedings of the 39th IMAC, A Conference and Exposition on Structural Dynamics 2021.

[65] Malekjafarian A., OBrien E.J. (2017). On the use of a passing vehicle for the estimation of bridge mode shapes. J. Sound Vib..

[66] Da Marulan J., Caicedo J.M., Thomson P. (2017). Mode shapes identification under harmonic excitation using mobile sensors. Ing. Y Competividad.

[67] Li Z.H., Au F.T.K. (2014). Damage Detection of a Continuous Bridge from Response of a Moving Vehicle. Shock. Vib..

[68] Khorram A., Bakhtiari-Nejad F., Rezaeian M. (2012). Comparison studies between two wavelet based crack detection methods of a beam subjected to a moving load. Int. J. Eng. Sci..

[69] McGetrick P., Kim C.-W. (2013). A Parametric Study of a Drive by Bridge Inspection System Based on the Morlet Wavelet. Key Eng. Mater..

[70] O’Brien E.J., McGetrick P., González A. (2014). A drive-by inspection system via vehicle moving force identification. Smart Struct. Syst..

[71] Zhu X.Q., Law S.S., Huang L., Zhu S.Y. (2018). Damage identification of supporting structures with a moving sensory system. J. Sound Vib..

[72] Locke W., Sybrandt J., Redmond L., Safro I., Atamturktur S. (2020). Using drive-by health monitoring to detect bridge damage considering environmental and operational effects. J. Sound Vib..

[73] Malekjafarian A., Golpayegani F., Moloney C., Clarke S. (2019). A Machine Learning Approach to Bridge-Damage Detection Using Responses Measured on a Passing Vehicle. Sensors.

[74] Elhattab A., Uddin N., OBrien E. (2016). Drive-by bridge damage monitoring using Bridge Displacement Profile Difference. J. Civ. Struct. Health Monit..

[75] Keenahan J., O’Brien E.J. Allowing for a rocking datum in the analysis of drive-by bridge inspections. Proceedings of the Civil Engineering Research in Ireland.

[76] OBrien E.J., Keenahan J. (2015). Drive-by damage detection in bridges using the apparent profile. Struct. Control Health Monit..

[77] Quirke P., Bowe C., OBrien E.J., Cantero D., Antolin P., Goicolea J.M. (2017). Railway bridge damage detection using vehicle-based inertial measurements and apparent profile. Eng. Struct..

[78] Yin S.-H., Tang C.-Y. (2011). Identifying Cable Tension Loss and Deck Damage in a Cable-Stayed Bridge Using a Moving Vehicle. J. Vib. Acoust..

[79] Malekjafarian A., McGetrick P.J., OBrien E.J. (2015). A Review of Indirect Bridge Monitoring Using Passing Vehicles. Shock. Vib..

[80] Shokravi H., Shokravi H., Bakhary N., Heidarrezaei M., Rahimian Koloor S.S., Petrů M. (2020). Vehicle-Assisted Techniques for Health Monitoring of Bridges. Sensors.

[81] Yang Y.B., Yang J. (2017). State-of-the-Art Review on Modal Identification and Damage Detection of Bridges by Moving Test Vehicles. Int. J. Struct. Stab. Dyn..

[82] Fitzgerald P.C., Malekjafarian A., Cantero D., OBrien E.J., Prendergast L.J. (2019). Drive-by scour monitoring of railway bridges using a wavelet-based approach. Eng. Struct..

[83] Iwnicki S. (1999). The Manchester Benchmarks for Rail Vehicle Simulation.

[84] Adhikary S., Singh Y., Paul D.K. (2014). Modelling of soil-foundation-structure system. Soil Dyn. Earthq. Eng..

[85] Micu E.A., OBrien E.J., Bowe C., Fitzgerald P., Pakrashi V. (2022). Bridge Damage and Repair Detection Using an Instrumented Train. J. Bridge Eng..

[86] Clough R.W., Penzien J. (1993). Dynamics of Structures.

[87] Zhai W.M., Wang K.Y., Lin J.H. (2004). Modelling and experiment of railway ballast vibrations. J. Sound Vib..

[88] Kwon Y.W.B.H. (1997). The Finite Element Method Using MATLAB.

[89] Yang Y.B., Yau J.D., Wu Y.S. (2004). Vehicle-Bridge Interaction Dynamics—With Applications to High-Speed Railways.

[90] Fitzgerald P.C. (2019). University College Dublin. School of Civil, E. A Vibration-Based Approach to Bridge Structure and Substructure Condition Monitoring Using Direct and Drive-by Methods. Ph.D. Thesis.

[91] Robertson I.N., Riggs H.R., Yim S.C.S., Young Y.L. (2007). Lessons from hurricane Katrina storm surge on bridges and buildings. J. Waterw. Port Coast. Ocean. Eng..

[92] Prendergast L.J., Gavin K. (2016). A comparison of initial stiffness formulations for small-strain soil-pile dynamic Winkler modelling. Soil Dyn. Earthq. Eng..

[93] Zanini M., Faleschini F., Ademovic N., Prendergast L., Gavin K., Limongelli M.P. Structural health monitoring and design code compliance for performance assessment of bridges under scour and seismic hazards. Proceedings of the Value of Structural Health Monitoring for the Reliable Bridge Management.

